# Supplementary material for: U.S. West Coast droughts and heat waves exacerbate pollution inequality and can evade emission control policies
Source: Nat Commun. 2023 Mar 23;14:1415. doi: 10.1038/s41467-023-37080-0 (PMC10036627; doi:10.1038/s41467-023-37080-0)
Supplement: Supplementary file 1 — Supplementary Information [file 41467_2023_37080_MOESM1_ESM.pdf]

Supplemental Information

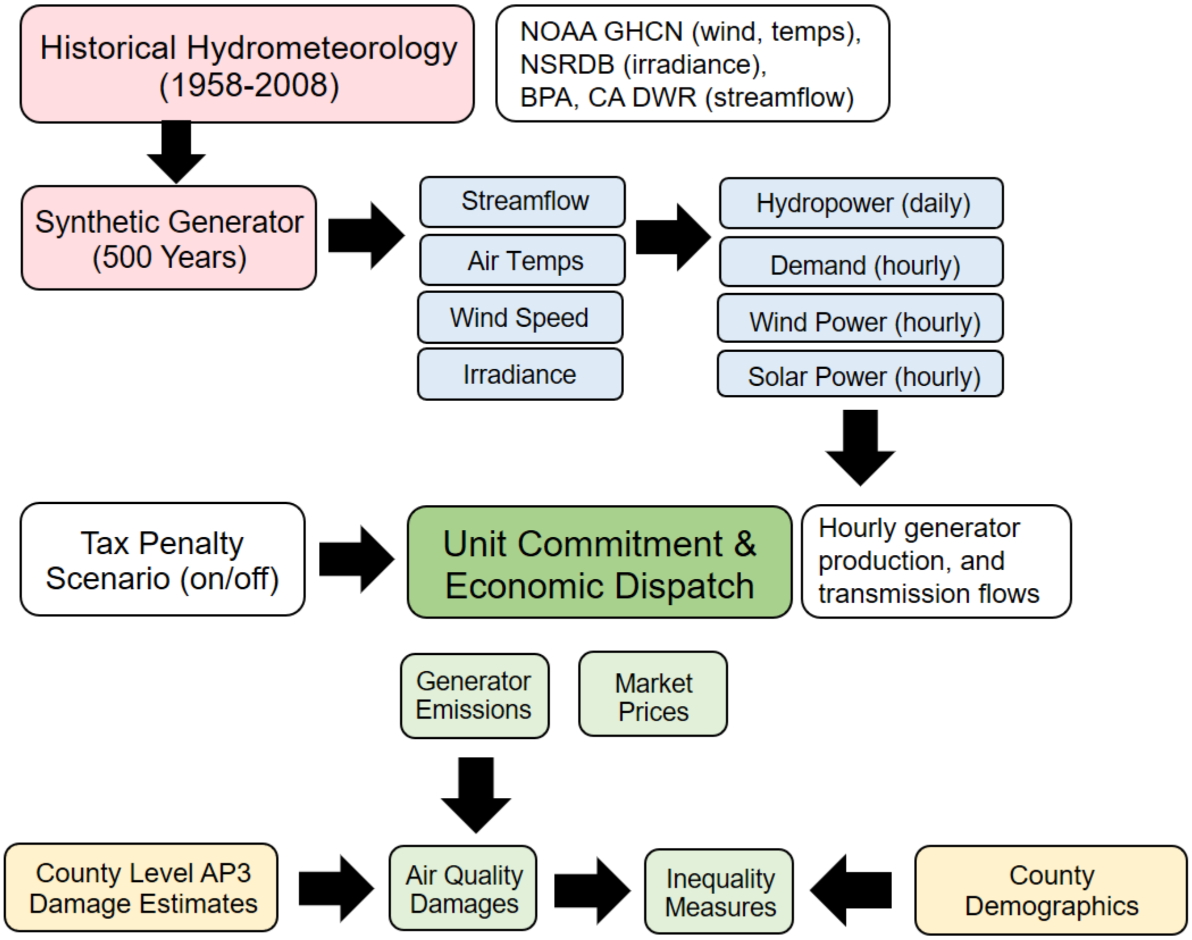

Figure S1. CAPOW's workflow. The stochastic engine takes the historical hydrometeorological data (streamflow, air temperature, wind speed, and solar irradiation) from different sources and transforms them into 500 years of synthetic hydrometeorological data and then to the power system related inputs (available hydropower, solar power, wind power, and electricity demand). Then it runs the UC/ED model under various tax scenarios. The model output is the hourly power generation of each power plant in CAISO, the wholesale market price, which later on translates into the individual power plants' air pollution emissions and air pollution. Then using the county demographics, air pollution damages gets translated to inequality measures.

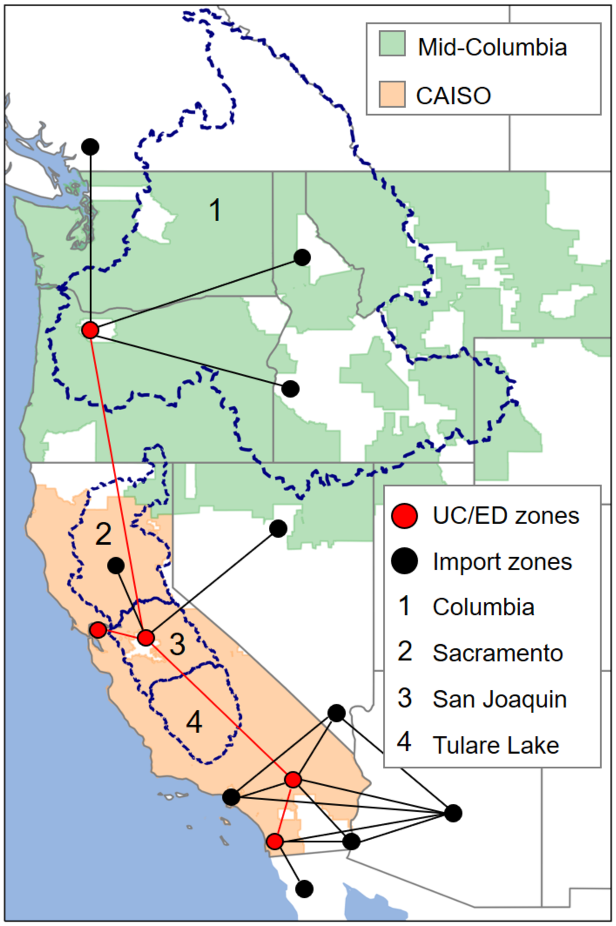

Figure S2. Topology of CAPOW's model. The geographical scope of CAPOW is consists of two major wholesale electricity markets (Mid-Columbia (Mid-C) market (green area) in the Pacific Northwest and the California Independent System Operator (CAISO) in California (orange area)) and 4 major river basins (1-Columbia river, 2-Sacramento river, 3-San Joaquin river, and 4-Tulare Lake).

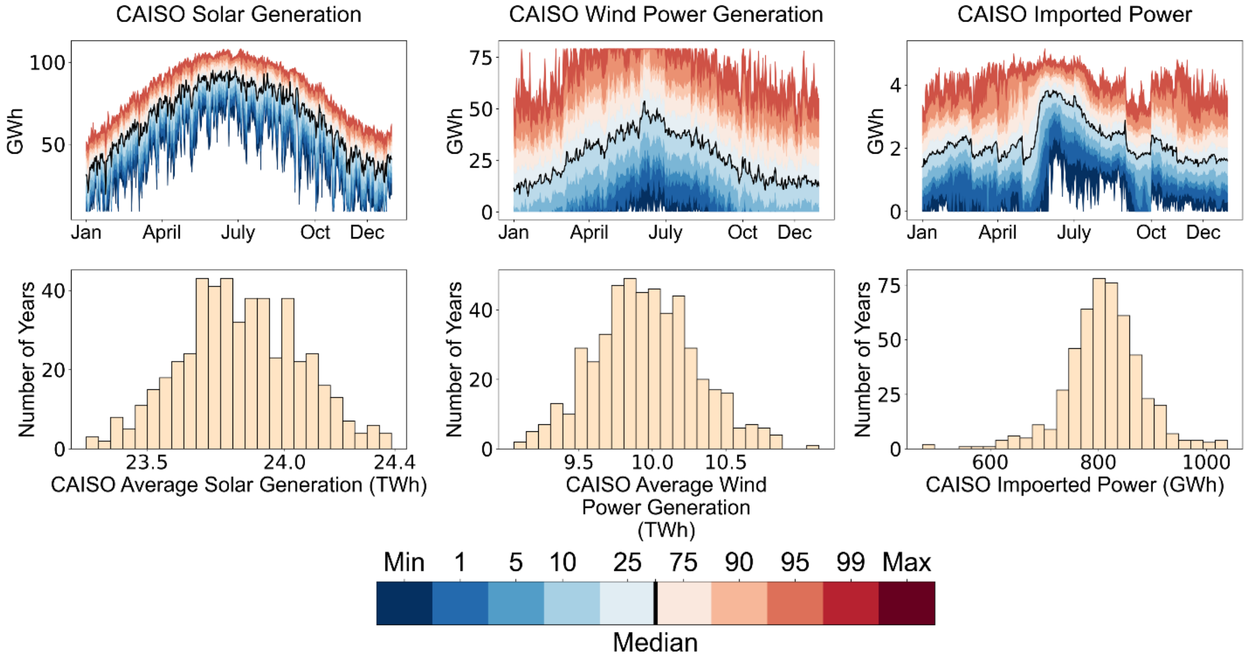

Figure S3. Simulated annual and seasonal uncertainty in solar and wind power generation and imported power from PNW.

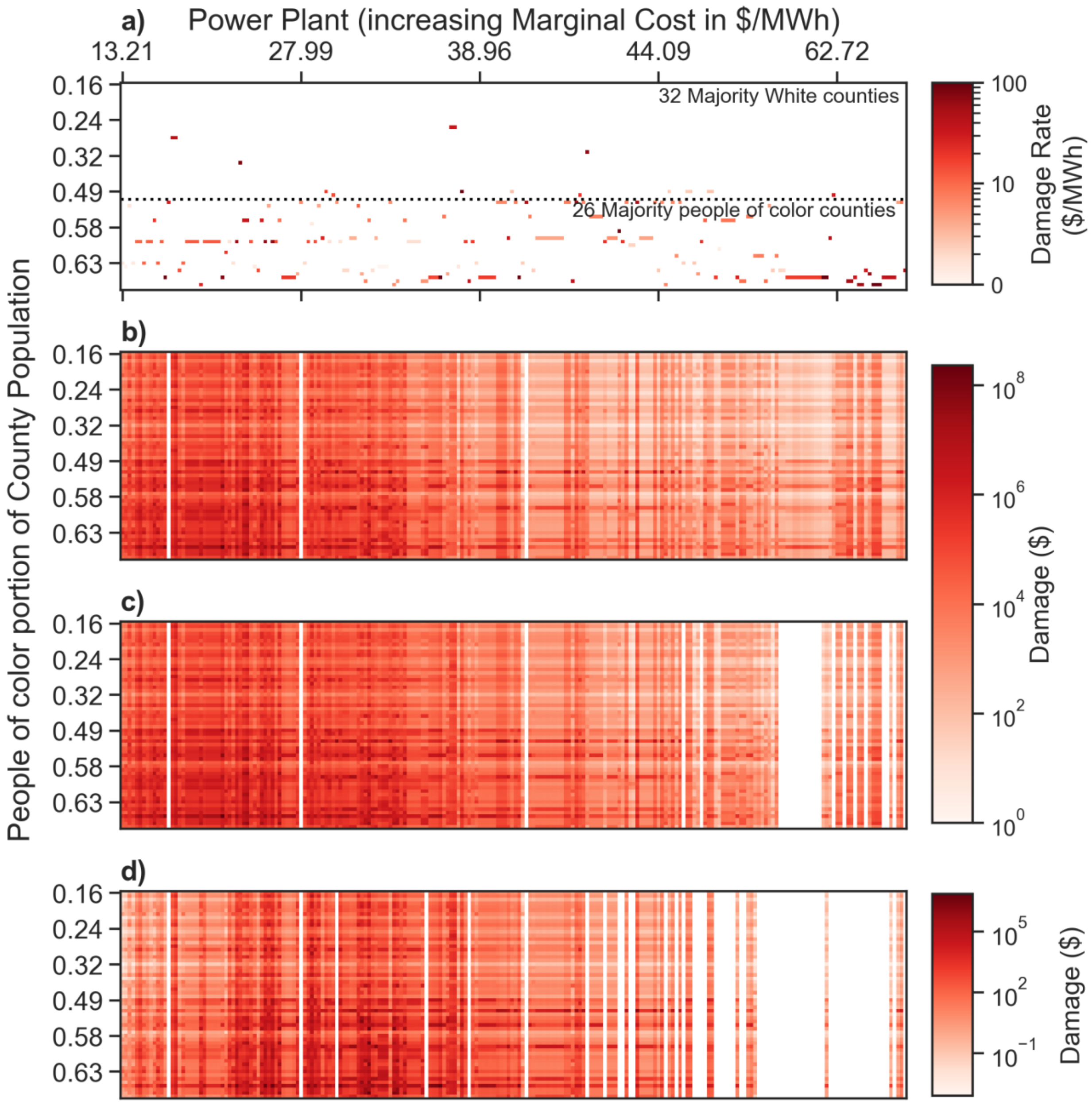

Figure S4. The panels in this plot show the power plants on the x-axis (sorted by their marginal price of power generation) and CA counties on the y-axis (sorted by portion of people of color population). Panel "a" shows the damage rate (which is equal to the generator's local emission tax) for each. This panel shows that most power plants are located in majority people of color counties. The colors on the next panels show the average air pollution damages (panel b), worst year air pollution damages (panel c), and the difference between the worst year and the average air pollution damages (panel d) from each generator to each county.

Figure S4 above provides additional information about how drought and extreme heat exacerbate existing pollution inequalities. All four panels (a-d) show information about power plants in the CAPOW model in 2D matrix form. Each row contains information about a specific county. Counties are sorted from top to bottom according to the percentage of each county's population that identifies as people of color. Each column contains information about a specific power plant. Power plants are sorted from left to right according to each power plant's marginal cost (in $/MWh).

In panel a), the colored pixels represent logical (x,y) ordered pairs of (county, power plant). A colored pixel means that a power plant (y) does exist in county x. The color signifies the air pollution damage rate caused by that power plant across all counties in CA (this is the same quantity as the local air tax identified for each power plant), with blue representing low damages per MWh produced, and red representing high. The vast majority of power plants in California is located in the 26 (out of a total of 58) people of color majority (i.e. >50%).

In panel b) the colors of each pixel instead signify the median air pollution damage (in $) delivered from power plant (y) to county (x) across the 500-year stochastic simulation. In general, each column (whether largely blue or red) shows a noticeable, red-ward shift (indicating a significant increase in air pollution damages) at the precise point on the y-axis where county demographics shift from majority White population to majority people of color.

In panel c) the colors of each pixel instead signify air pollution damage (in $) delivered from power plant (y) to county (x) in the year with the highest annual damages across the 500-year stochastic simulation. Again, we see a noticeable, red-ward shift (indicating a significant

increase in air pollution damages) at the precise point on the y-axis where county demographics shift from majority White population to majority people of color. Taken together, this information strongly supports the finding that people of color counties are disproportionately impacted by power plant air pollution in both "normal" and extreme years.

Panel d) shows the difference between panel b) and panel c). In other words, it shows the impacts of large negative anomalies in streamflow (drought) and large positive anomalies in air temperatures associated experienced during the year with the highest annual damages. Again, we see the red-ward shift in the bottom half of the panel. This indicates that the increased damages associated with grid scarcity also disproportionately impact people of color majority counties.

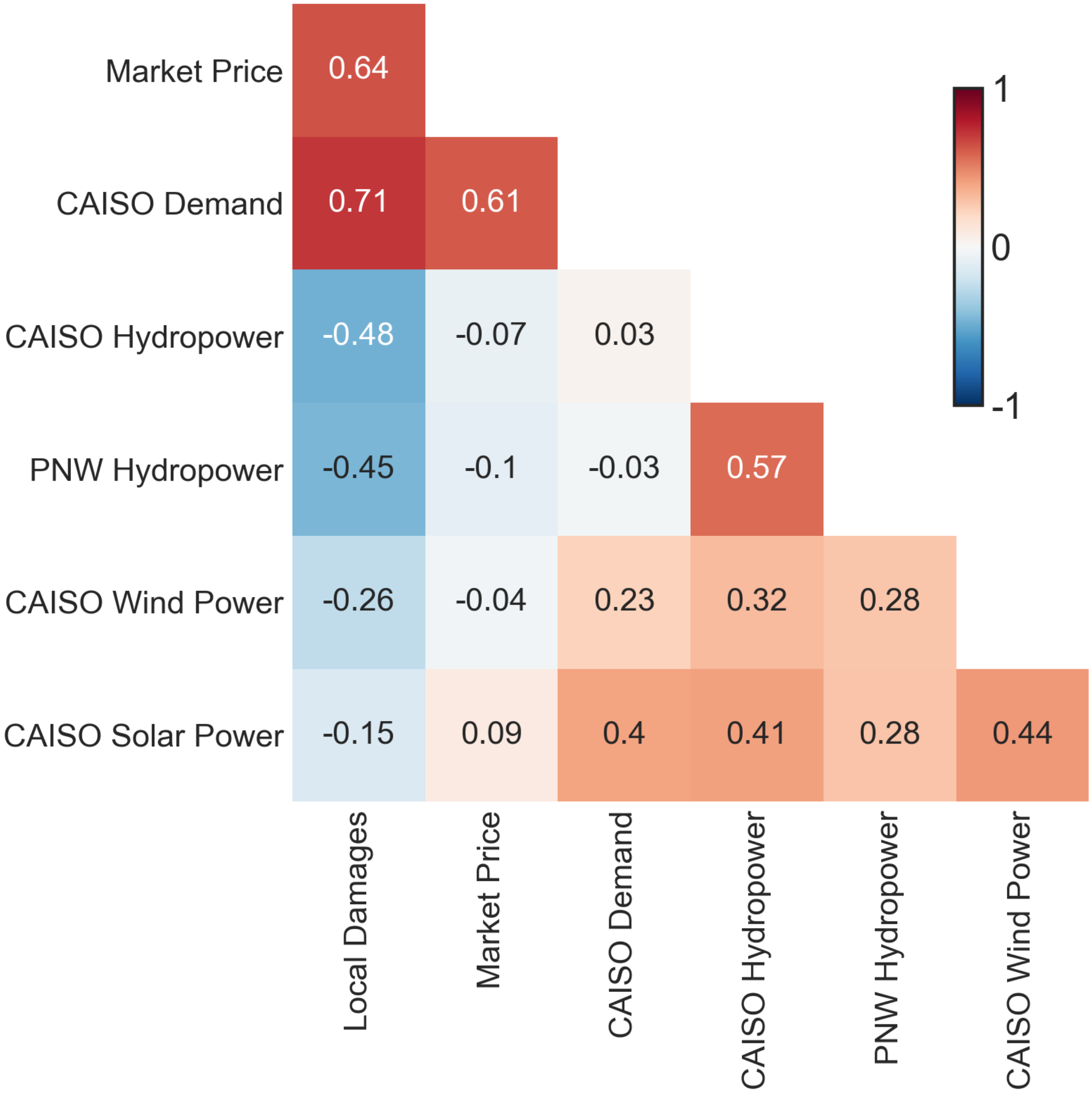

Figure S5. Daily correlations between various system performance metrics and state variables under the No Tax scenario. On a daily basis, there is a more positive correlation between air pollution damages and electricity demand and also a less negative correlation between air pollution damages and CAISO hydropower availability.

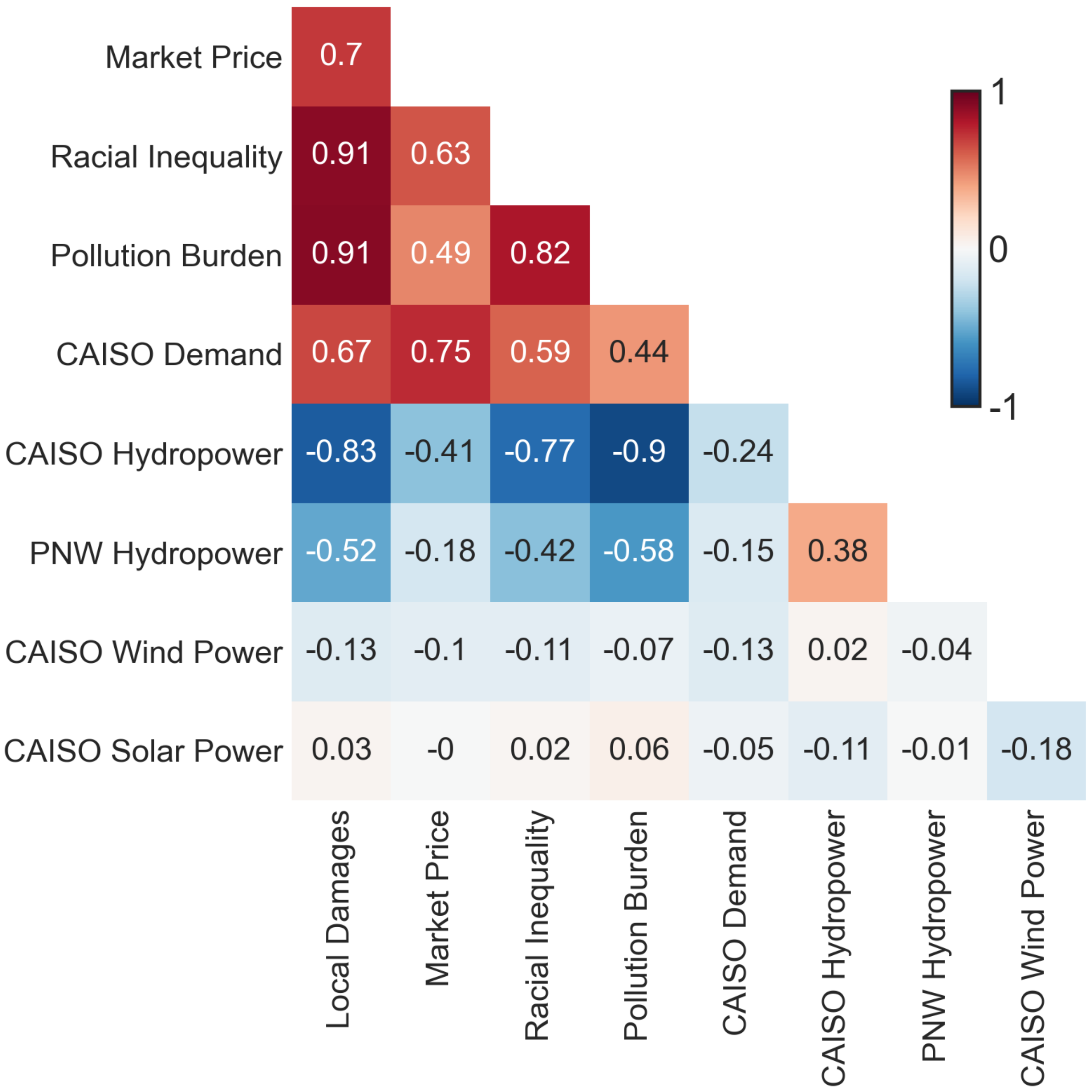

Figure S6. Annual correlations between various system performance metrics and state variables under the local emissions tax scenario.

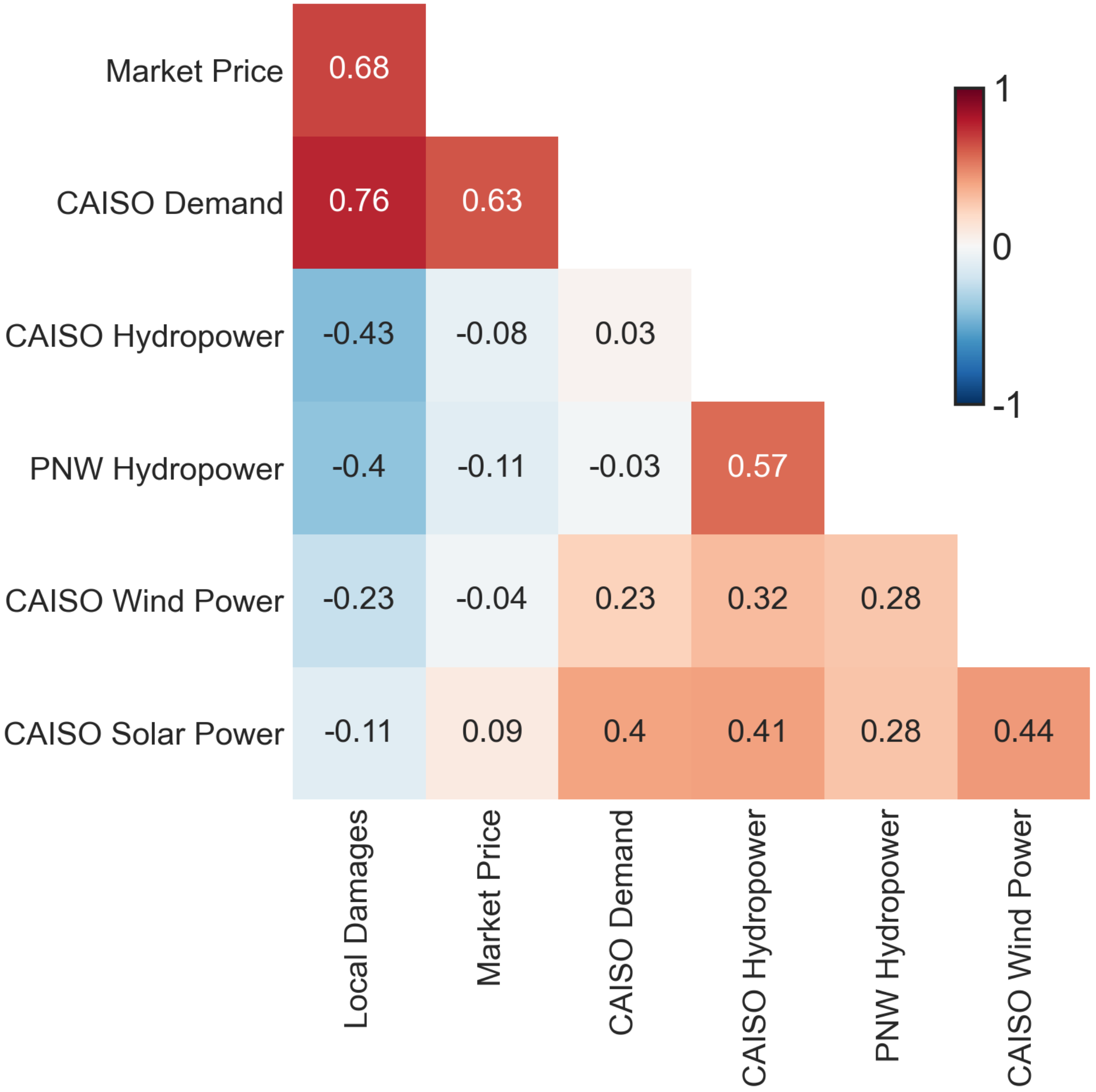

Figure S7. Daily correlations between various system performance metrics and state variables under the local emissions tax scenario. There are minimal differences in the daily correlation matrices of the no tax and local tax scenario.

The influence of penalties on power plant emissions

A key unanswered question that this study seeks to answer is whether heat waves and drought undermine the effectiveness of air pollution penalties that are put in place to reduce health damages. Our model simulations include the exploration of 4 different emissions control policies: 1) a base case (no penalties on either local air pollutants or CO_2 emissions; 2) a local air pollution tax (penalties on generator specific damages from emissions of SO_2, NO_X, and $PM_{2.5}$); 3) a CO_2 tax (penalties on generator specific damages from emissions of CO_2); and 4) a combined tax scenario in which a tax on local air pollutants and CO_2 emissions are enacted simultaneously. In the main paper, we only directly discuss results for the base case and local tax scenario. Our rationale for limiting results and discussion to these scenarios is that inclusion of penalties on CO_2 emissions, at least in the "short term" (without considering changes in the actual capacity mix), primarily serves to increase market prices without having a meaningful effect on air pollution damages from SO_2, NO_X and $PM_{2.5}$ (Figure S8).

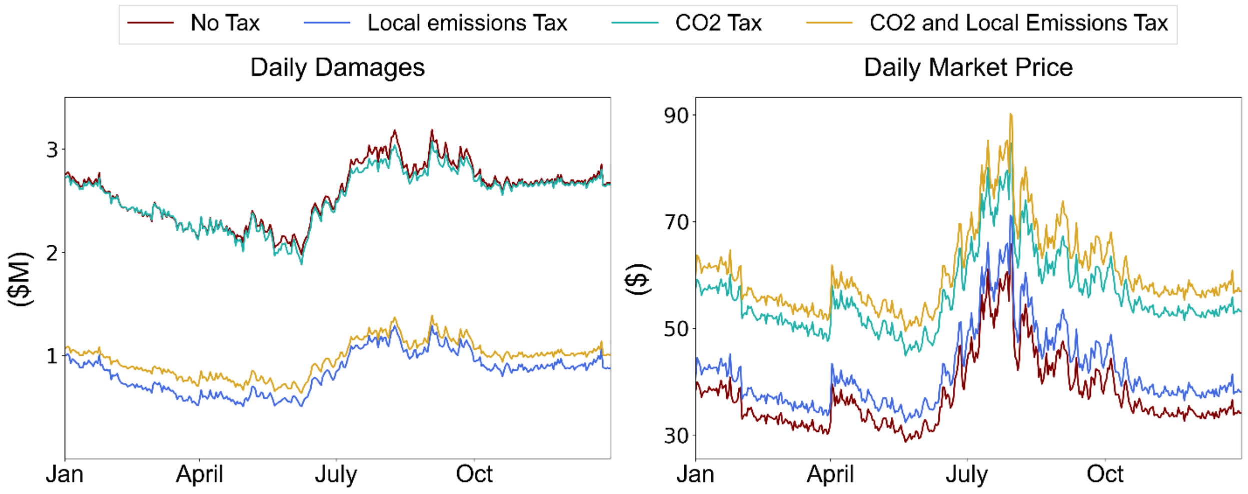

Figure S8. Daily average air pollution damages and market prices under various penalty scenarios calculated for the 500-year stochastic ensemble. Although the local tax scenario reduces the average air pollution damage over the 500 year ensemble by 67%, it only increases the average market price by 11%.

As expected, implementing penalties on power plant emissions of SO_2, NO_X, and $PM_{2.5}$ under the local tax scenario leads to a significant (69% on average) reduction in annual damages across the 500-year ensemble relative to the base case (Figure S9). Power plants whose emissions inflict higher health damages are penalized more heavily are utilized less often by the modeled CAISO operator according to its cost-minimizing objective.

Also, note that in Figure S9 the presence of the local tax does not prevent damages of emissions from increasing to extremely high levels on certain days. This hints at an answer to the central question of whether droughts and heat waves undermine the effectiveness of emissions penalties.

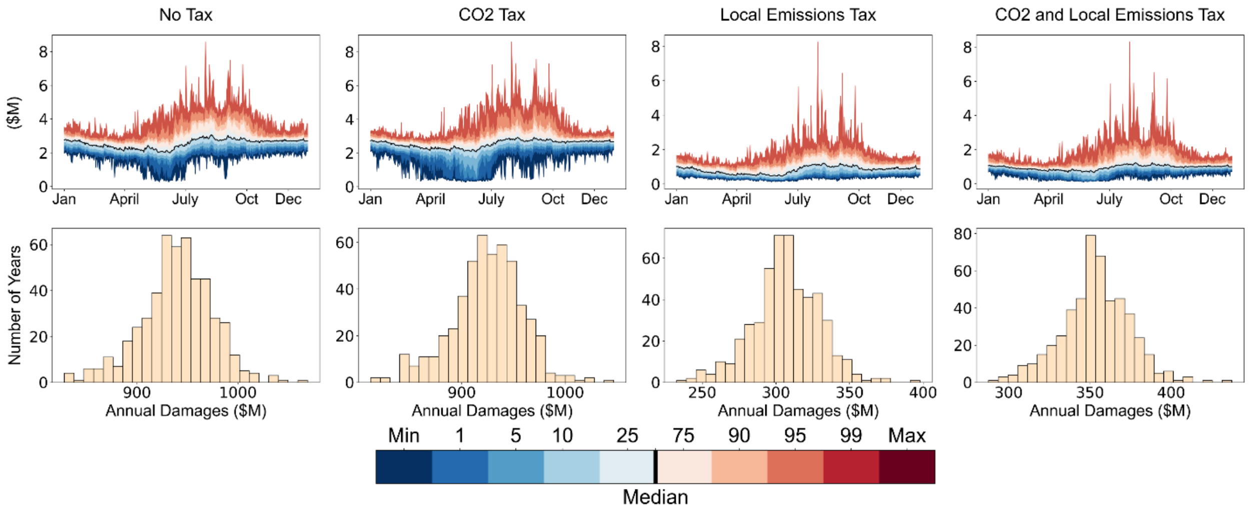

Figure S9. Simulated annual and seasonal uncertainty in total damages from local pollutants (SO_2, NO_X, and $PM_{2.5}$) under various tax scenarios. It should be noted that the peak on all of the four panels of the first row is the highest air pollution damage day observed in Figure 2, Figure 4, Figure 5, and Figure 6. None of the tax scenarios effectively reduce the air pollution damages on this specific day. Otherwise, the local tax is an effective policy at reducing air pollution damages.

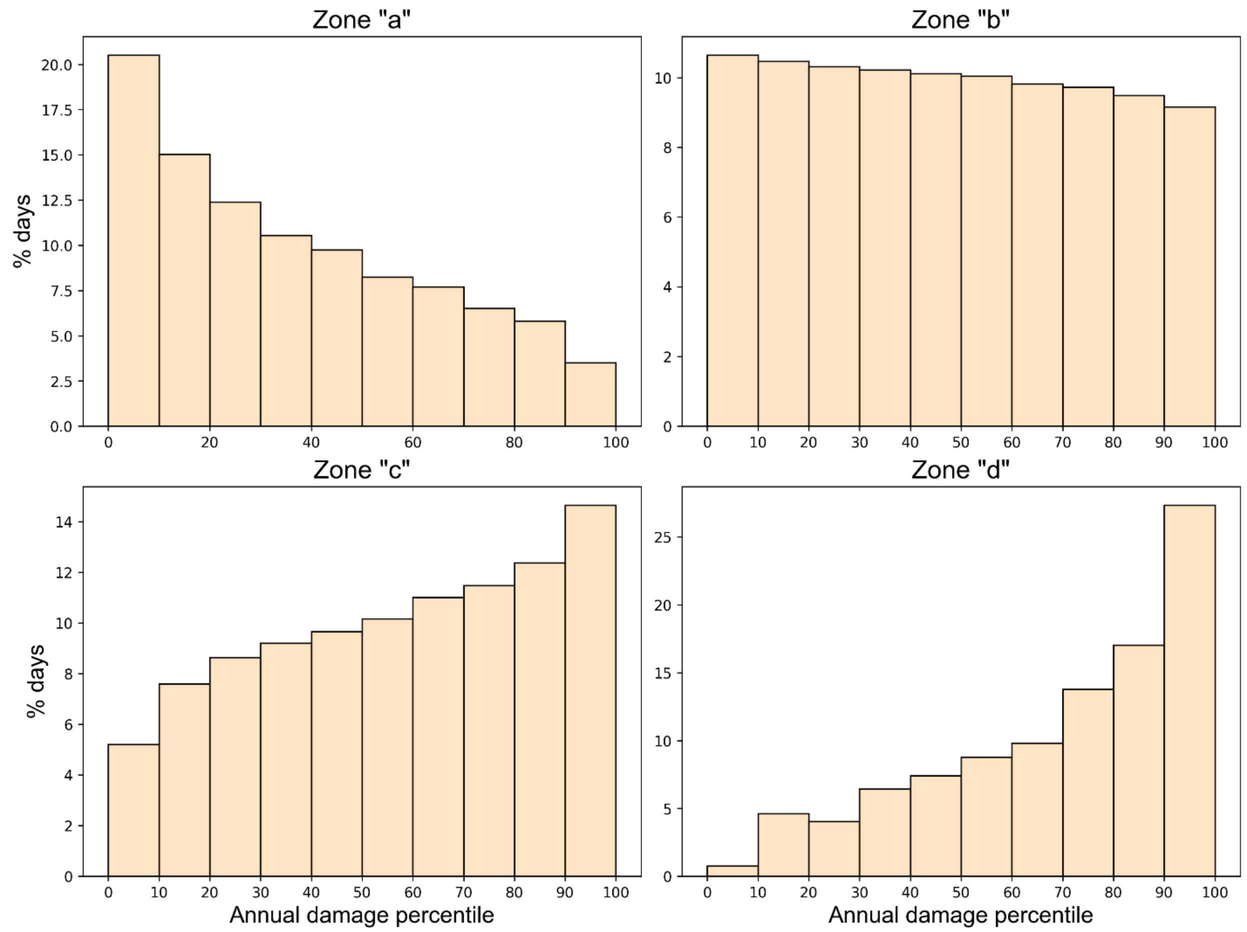

Figure S10. This figure shows the distribution of days in each zone of Figure 5 based on the days corresponding year's annual damages. It shows "bad days" (Zone "d") can happen in any year, but it is more probable to see a "bad day" in a "bad year".

A paradoxical effect of the local air tax on pollution inequality

An unanticipated finding from our experiment is that while taxing emissions of SO_2, NO_X and $PM_{2.5}$ dramatically reduces overall air pollution damages (and reduces damages most for people of color communities on a per capita basis), it simultaneously *strengthens* the relationship between drought and inequality in human health damages from local air pollutants inequality (R = -0.57 without a tax, R = -0.77 with local emissions tax). At an annual time step, the availability of hydropower in the CAISO system becomes more predictive of racial inequality and inequality in pollution burden (positively correlated with both). Damages under an local tax scenario are much lower overall, but could potentially be more concentrated in majority people of color counties and counties with higher pre-existing pollution burdens.

To understand why, we must investigate what happens at the individual generator level. Figure S11 shows modeling results for each power plant (circle) over the full 500-year ensemble. The size of each circle corresponds to installed capacity, while color signifies the $/MWh damages caused by each power plant's emissions of local air pollutants. The placement of each generator along the x-axis corresponds to its rank order marginal cost, with low cost generators on the left, and high cost generators on the right. The top row shows modeled results without a local air pollution tax in place; the bottom row shows results with the tax in place. Note that the rank order marginal cost of generators in the bottom row (with the tax in place) considers the effects of the tax (i.e. each power plant's color) on marginal cost.

Figure S11a shows each generator's capacity factor without a tax in place; in general, the cheapest generators (box I) have the highest capacity factors (these provide baseload power). Many of these power plants are relatively small generators with high emission damages ($/MWh). In Figure S11b, note that the same generators (box II) show a weaker negative

correlation with CA hydropower generation; this is because these plants act as baseload generators in our model, even in wet years. Box III in Figure S11c shows the portion of damages from these same generators that affect people of color majority (>50%) counties.

The bottom shows results with the local air pollution tax in place. The lowest marginal cost generators likewise generally show the highest capacity factors, but now these generators are those with low air pollution penalties (dark blue in color). These are typically much larger capacity generators, and due to their size they exhibit stronger negative correlations between capacity factor and CA hydropower availability (i.e. they are used less during wet years). Although these are relatively low damage generators, they often result in high damages to the people of color majority counties. The result is that while per capita damages in people of color majority communities decreases more than in majority White communities, inequality becomes more strongly correlated with drought with the local air tax in place.

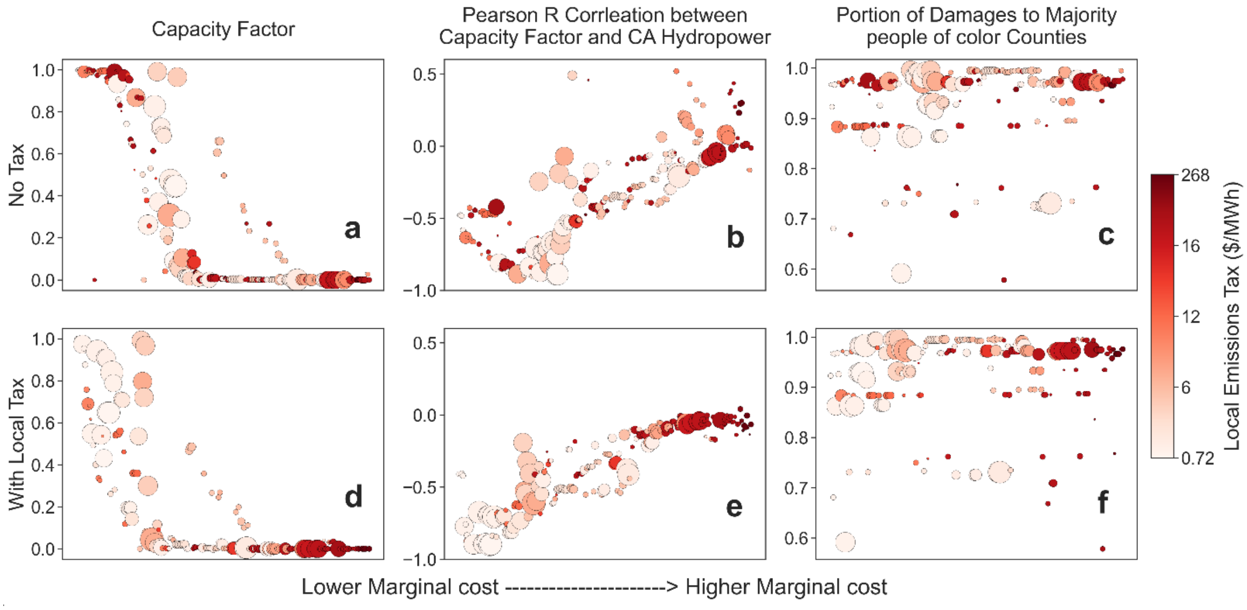

Figure S11. This figure shows the average properties of generators over the 500 year ensemble. The x-axis on all panels shows generators sorted by their marginal cost ($/MWh), without emission penalties (top row) and with the local tax (bottom row). The first column (panels a and d) show capacity factor, the middle column (panels b and e)

show the correlation between hydropower and power generation for all generators, and the column on the right (panels c and f) are the portion of damages that go to majority people of color counties)

Additional explanation of CAPOW

The stochastic engine that is used to create inputs to the CAPOW model produces synthetic hydrologic data (both streamflow and estimates of hydropower availability) on a daily time step. We then use publically available hydrologic mass balance models representing 85% of the hydropower capacity in the Pacific Northwest (versions of HYSSR, developed by the U.S. Army Corps of Engineers to simulate the Federal Columbia River Power System; and a ResSim model that simulates the operations of Federal dams in the Willamette River Basin). In California, we use hydrologic mass balance models to represent hydropower capacity at key state-run storage reservoirs (about 12% of total hydropower capacity). However, much of the state's hydropower capacity is privately owned and there is little information about the operation of these dams. Daily hydropower production at these projects is predicted using observed streamflow downstream. For each upstream hydroelectric dam, a corresponding downstream storage reservoir or stream gauge on the same river is identified. Dams are assumed to follow a different set of "operating rules" that translate observed downstream flows into estimates of upstream hydropower production. Rules are fitted using a differential evolution algorithm. About 15% of hydropower capacity in the Pacific Northwest and 20% of hydropower capacity in California are within the five core WECC zones that make up the UC/ED model but fall outside the four river basins mentioned above and are not associated with publically available models. These projects are modeled by scaling hydropower generation from nearby dams. Daily availabilities of hydropower then serve as inputs to the UC/ED module of CAPOW, which schedules hydropower production hourly as part of the cost minimizing mathematical program.

A more detailed description of how daily hydropower production is estimated, along with validation of historical and predicted hydropower generation can be found in Su et al. 2020[1].

Synthetic meteorological data (temperatures, solar irradiance, wind speeds) are also generated on a daily basis. We use this information to estimate daily values of peak electricity demand, daily solar and wind power production. Synthetic time series of heating and cooling degree days and associated wind speeds for all 17 GHCN stations are used as independent variables in multivariate regressions of daily peak electricity demand. Separate models were used for each of the five WECC zones that comprise the core UC/ED problem in CAPOW. The multivariate regressions were trained on historical weather and electricity demand data over the period 2010-2016. This approach is able to produce accurate estimates of daily peak electricity demand (R^2 values range from 0.75 to 0.89 across the five zones), with demand in zones that experience lower heating and cooling needs being more difficult to represent using temperatures and wind speeds alone. After simulating daily peak electricity demand for each zone, hourly electricity demand is determined by multiplying peak demands with 24-hour load profiles for each zone and each calendar day. These profiles, which are calculated using historical data, represent the typical fraction of daily peak demand experienced in each hour.

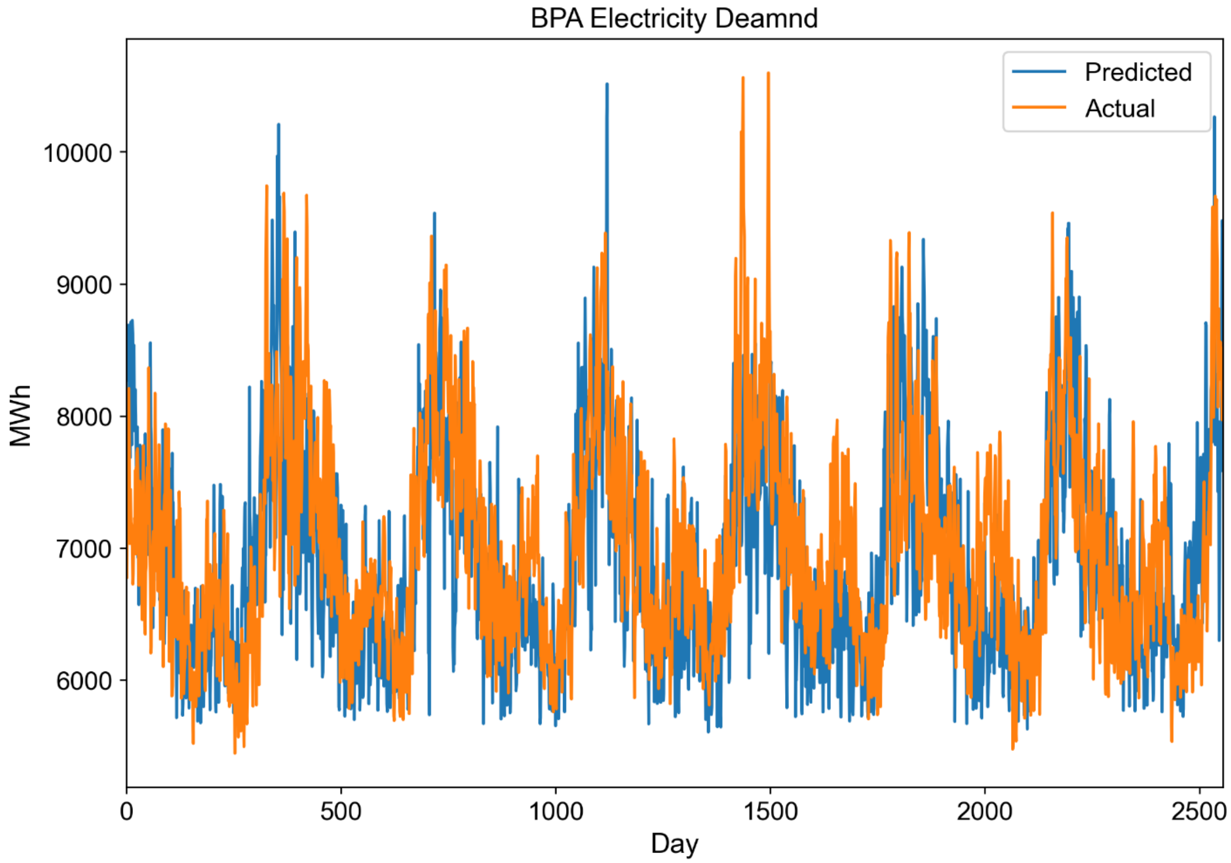

Figure S12. BPA Actual electricity demand for the 2010-2016 vs. CAPOW prediction of BPA electricity demand for the same period (R^2 = 0.89).

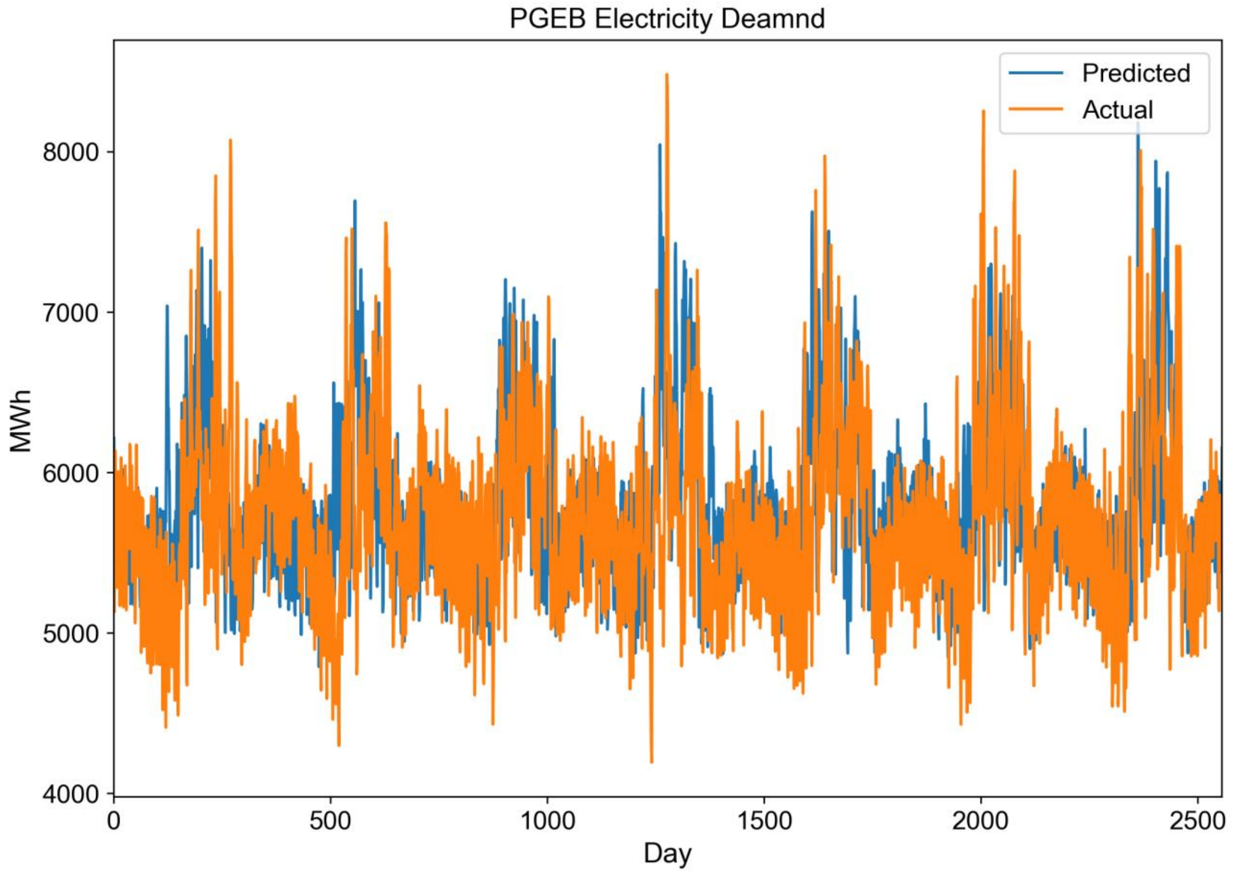

Figure S13. PGEB Actual electricity demand for the 2010-2016 vs. CAPOW prediction of BPA electricity demand for the same period (R^2 = 0.79).

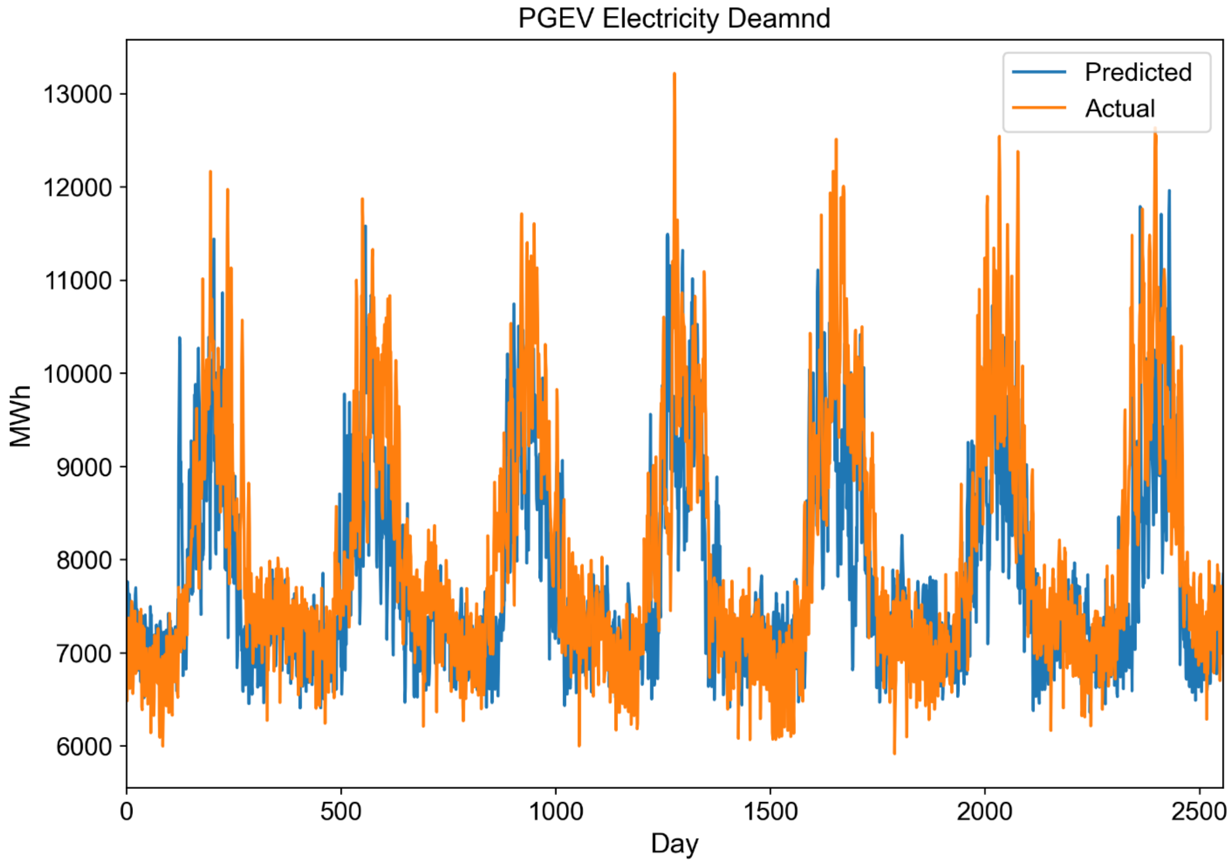

Figure S14. PGEV Actual electricity demand for the 2010-2016 vs. CAPOW prediction of BPA electricity demand for the same period (R^2 = 0.90).

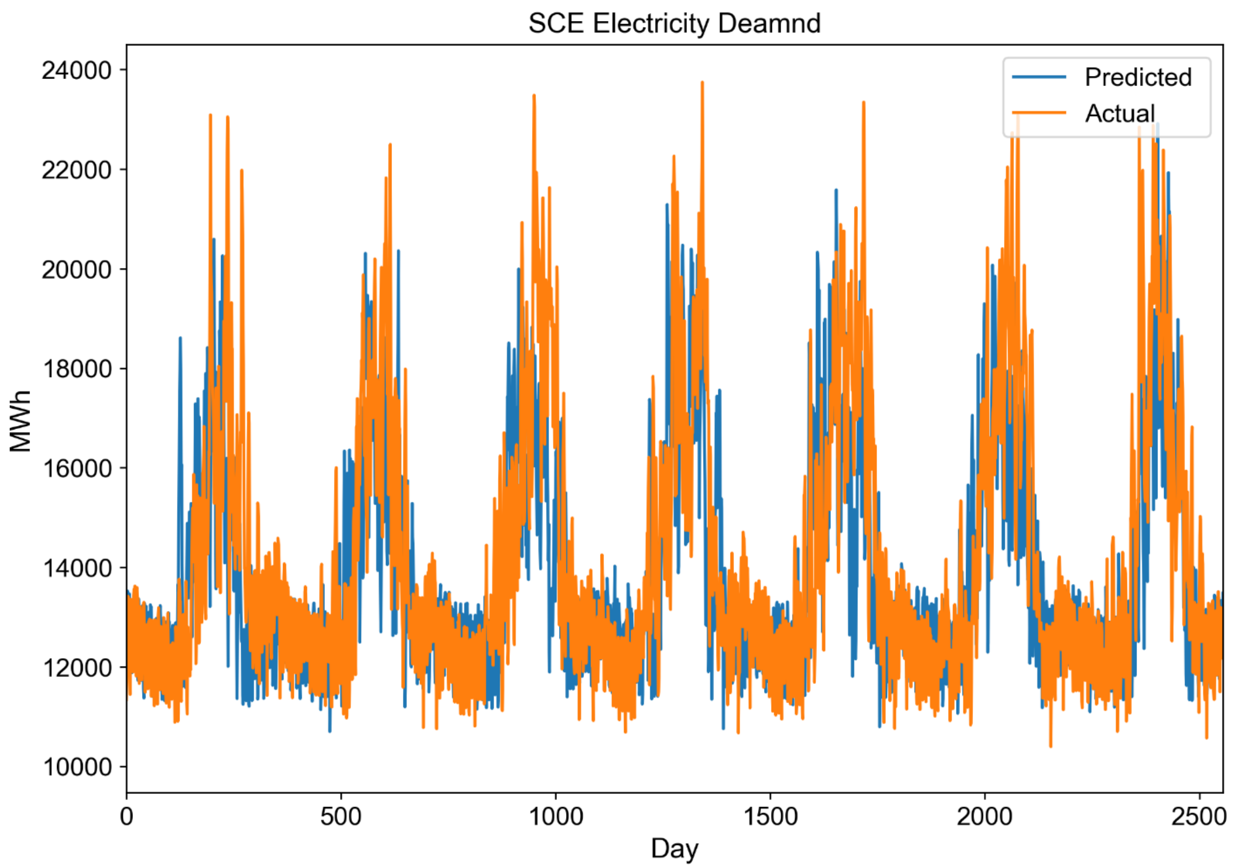

Figure S15. SCE Actual electricity demand for the 2010-2016 vs. CAPOW prediction of BPA electricity demand for the same period (R^2 = 0.89).

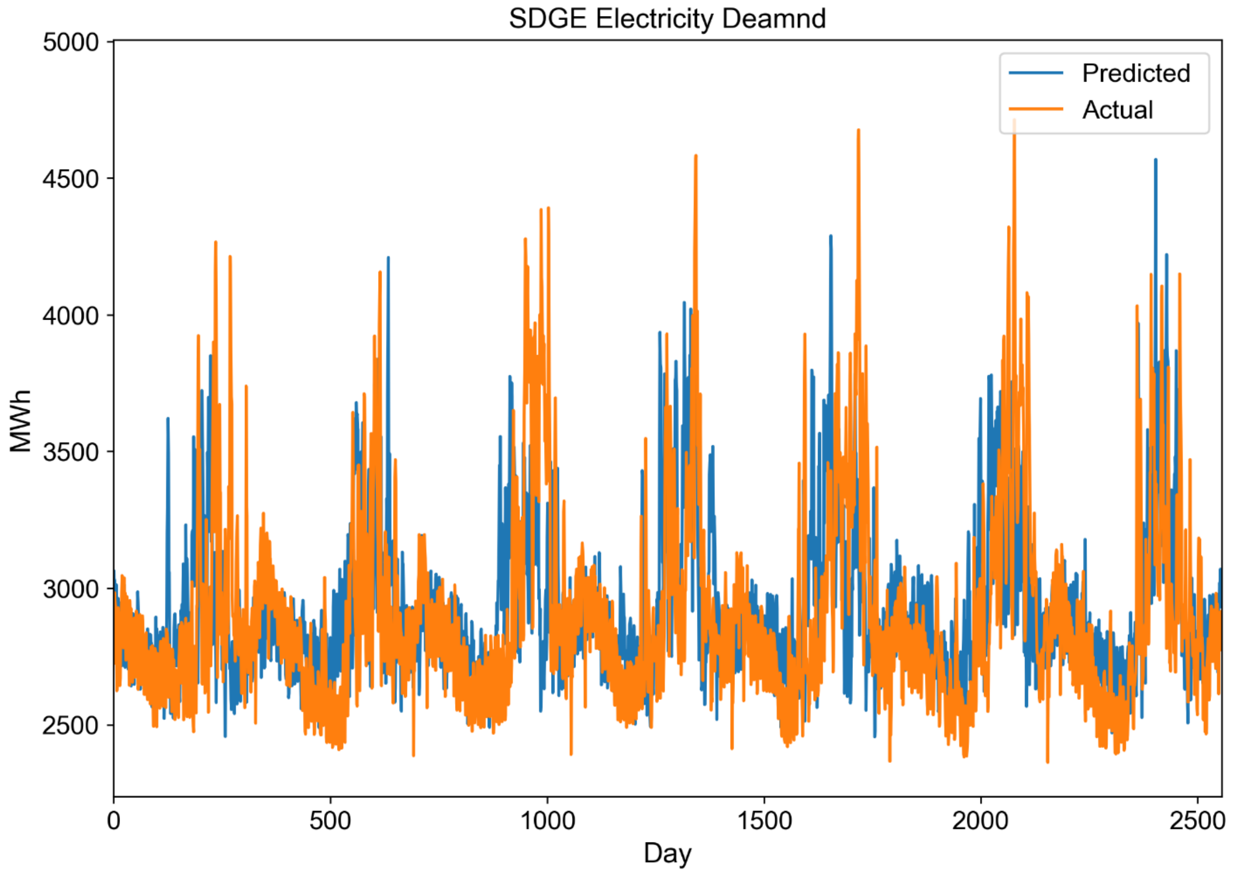

Figure S16. SDGE Actual electricity demand for the 2010-2016 vs. CAPOW prediction of BPA electricity demand for the same period (R^2 = 0.80).

The same weather data used to model electricity demand (see response to previous comment), along with daily values of hydropower production and wind power production in the Pacific Northwest zone of the CAPOW model, are used as independent variables in multivariate regressions that estimate exchanges of electricity between the five WECC zones that make up the core UC/ED model and neighboring WECC systems that are not currently represented mechanistically. Daily values of peak electricity demand, daily solar and wind power production are then disaggregated to an hourly time step using profiles sampled from historical hourly data. Historical data used as the basis for our synthetic generator are daily hydrometeorological data and hourly records of wind and solar power production and electricity demand.

Note that each year is generated by resampling from statistical distributions fitted to roughly 50 historic years and is independent from the rest of 500-year ensemble (i.e. the other 499 years). Within year, we go to very careful lengths to capture all relevant statistical dependencies in hydrometeorological processes (air temperatures, wind speeds, solar irradiance, streamflow). These include: seasonality, diurnal effects, daily and hourly autocorrelation, statistical moments, and cross correlations across variables and space. We also note that while our synthetic data successfully captures these dependencies, it does so while also producing combinatorial extremes (i.e. extremely high and low net load scenarios) that exist outside the limited historical record.

Imports/exports that are modeled statistically represent specific aggregated transmission pathways that WECC refers to by number. Historically, power flows on some of these WECC paths have been bi-directional; however, most often electricity flows along these paths are "imported" by the core UC/ED model (red circles in Figure S2) from an adjacent zone outside the core UC/ED model (black circles in Figure S2). "Exports" describe the opposite; a demand for electricity in an outside zone, which must be satisfied by generators within the core UC/ED model. Import/export regressions, are trained on observed daily path flow data (typically assuming the classification of: imports = positive flow values; exports = negative flow values). Path flow data are available for the years 2010-2012. Modeled imports/exports show R^2 values ranging from 0.77 to 0.95, suggesting that daily regional exchanges of electricity can be represented statistically using weather and streamflow data, which collectively drive zonal electricity demand and the availability of wind and hydropower.

Note: Because we model imports/exports as a function of hydrometeorological conditions, our approach for modeling these processes does inherently "respond" to grid scarcity in California, at least to the degree that we observed over the training period (2010-2012). For example, periods of high demand in CAISO are positively correlated with imports into California from the Pacific Northwest (PNW) and Southwest; this correlation is also preserved in our creation of synthetic imports/exports. However, exchanges between CAISO and other systems is not treated as a decision variable to be optimized. Instead, estimated exchanges are treated as a constraint on both the CAISO and separate PNW power systems model.

For an in depth explanation of the methods employed by the stochastic engine and data products created, please refer to Su et al. 2020[1].

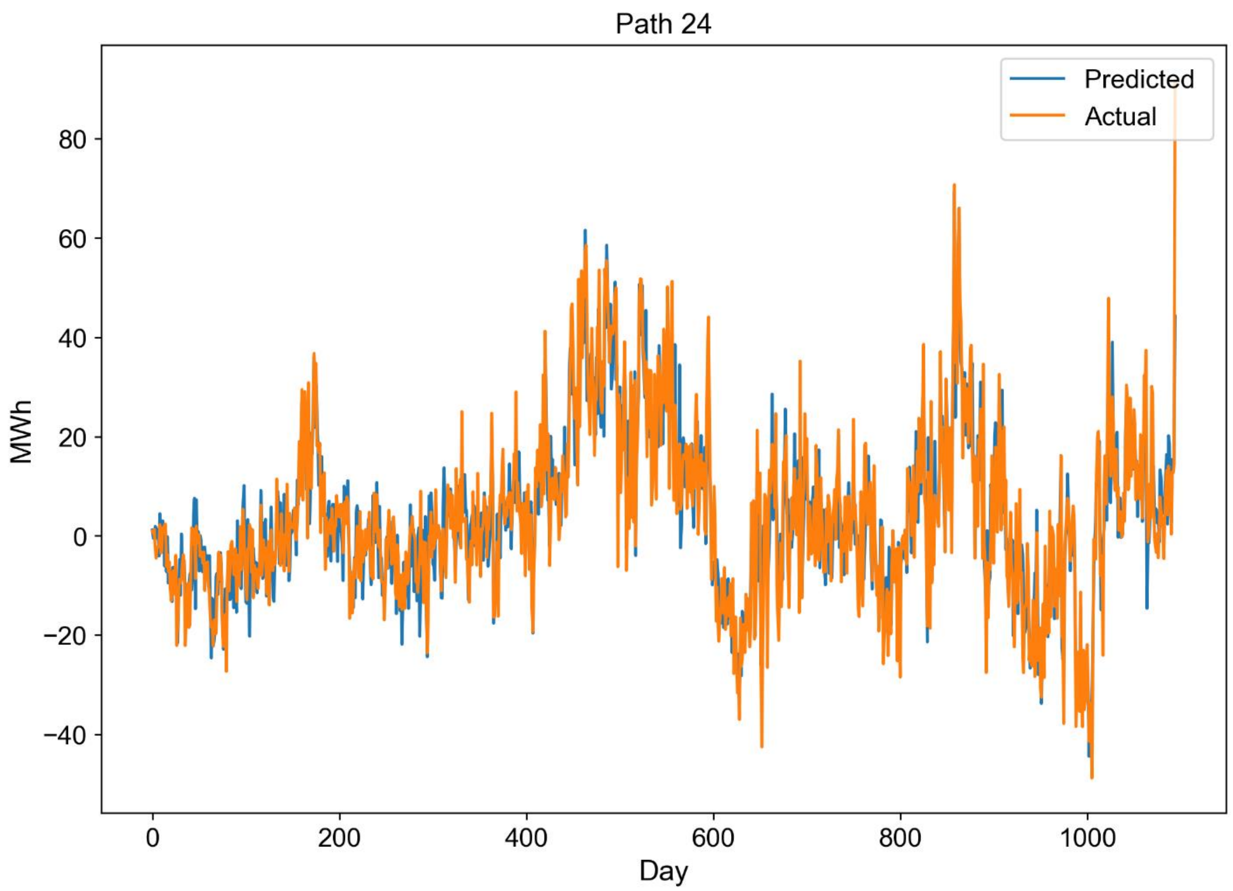

Figure S17. Actual power transfer through Path 24 for the 2010-2012 vs. CAPOW prediction of power flow for the same period (R^2 = 0.84)

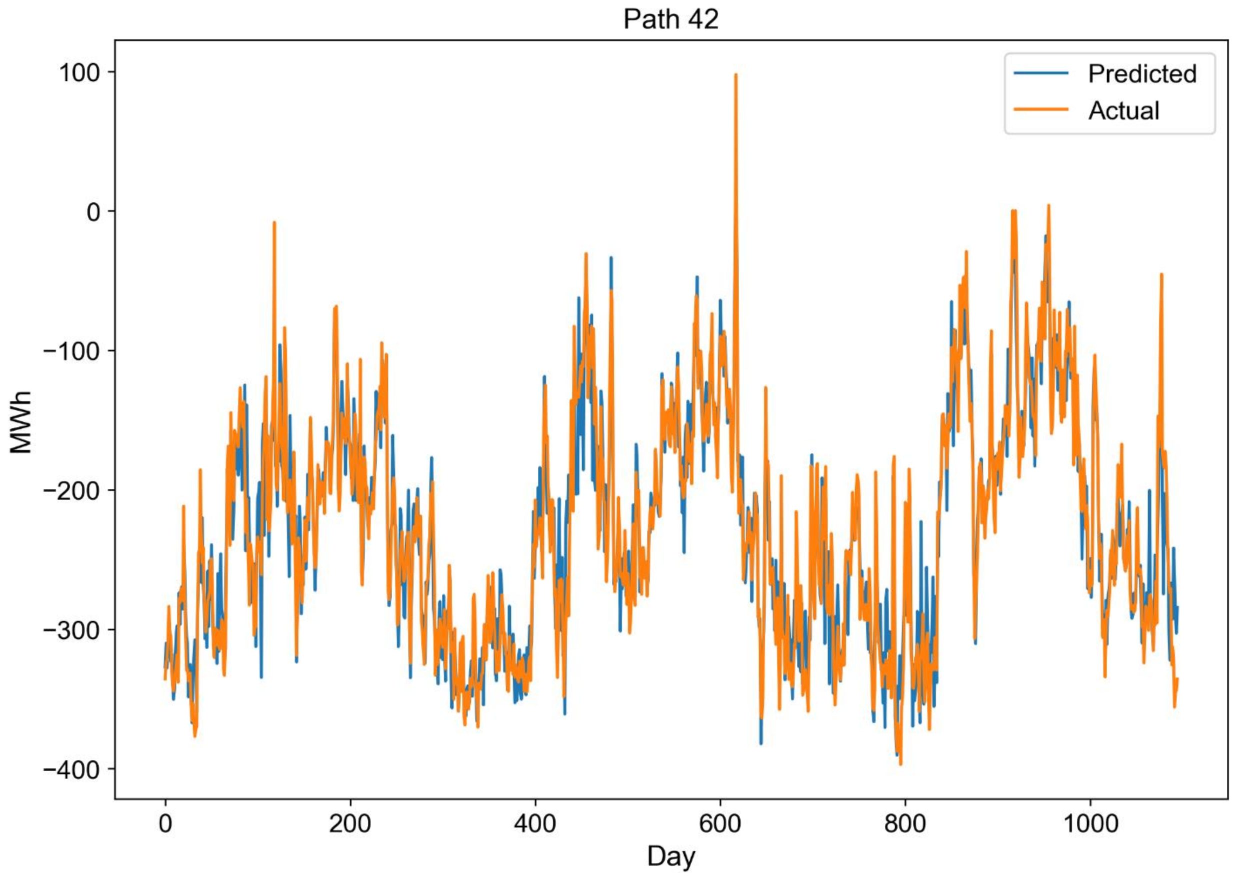

Figure S18. Actual power transfer through Path 42 for the 2010-2012 vs. CAPOW prediction of power flow for the same period (R^2 = 0.90)

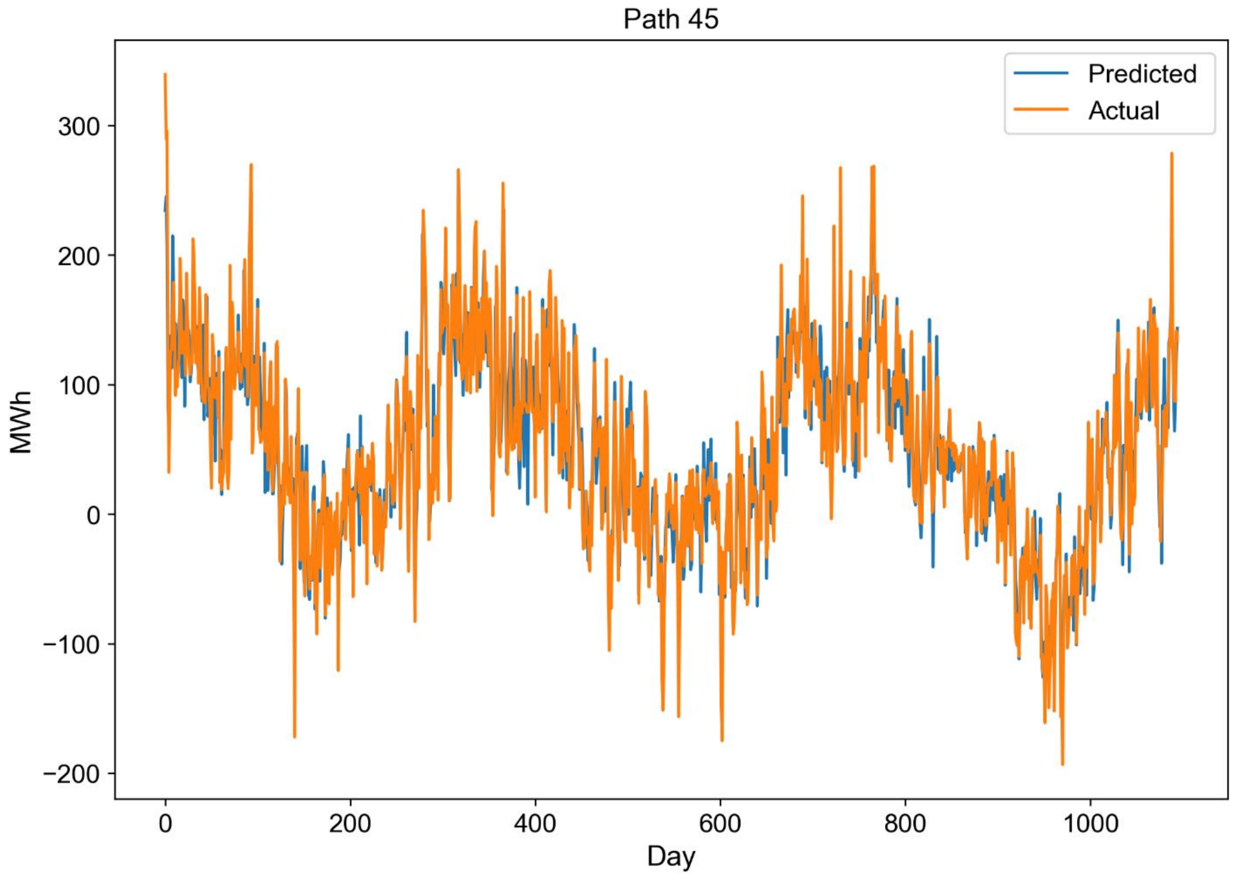

Figure S19. Actual power transfer through Path 45 for the 2010-2012 vs. CAPOW prediction of power flow for the same period (R^2 = 0.88)

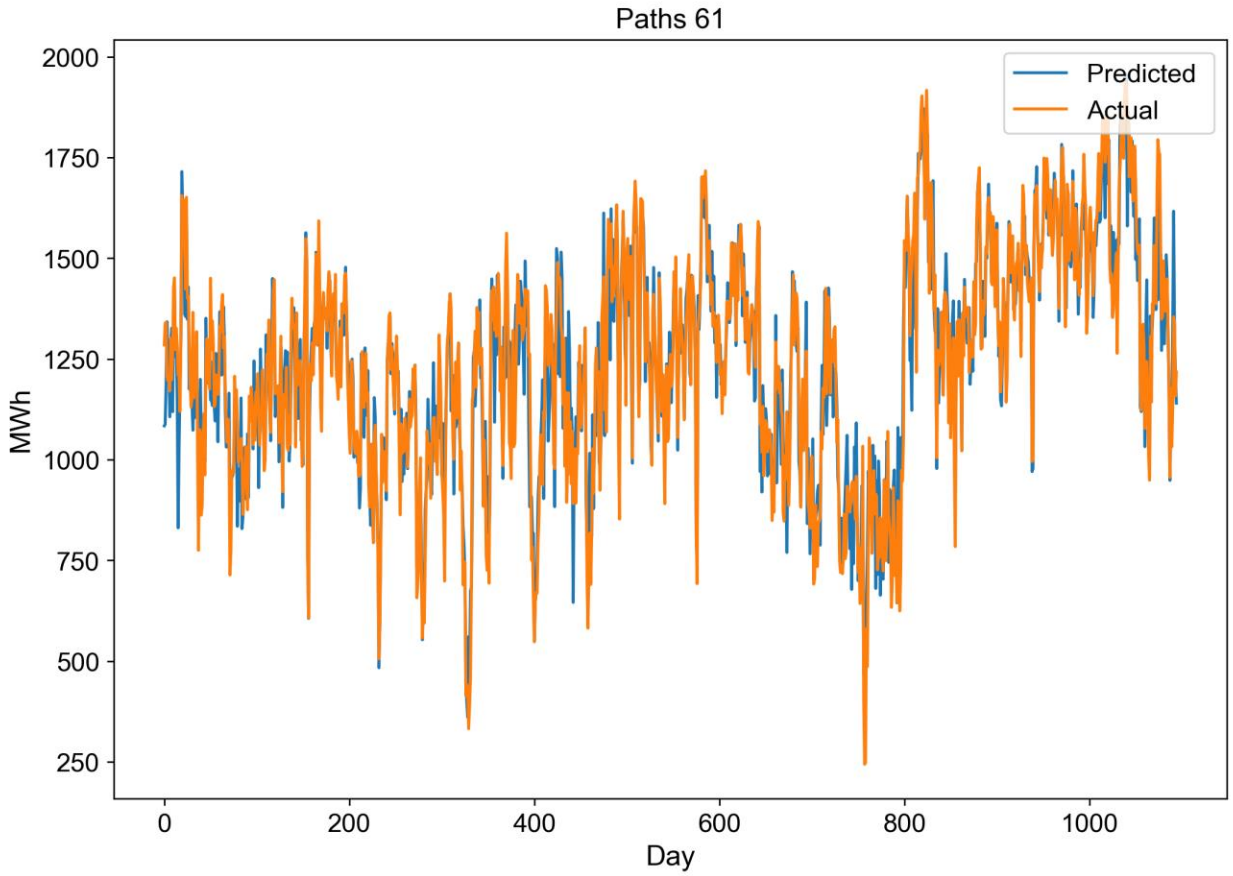

Figure S20. Actual power transfer through Path 61 for the 2010-2012 vs. CAPOW prediction of power flow for the same period (R^2 = 0.85)

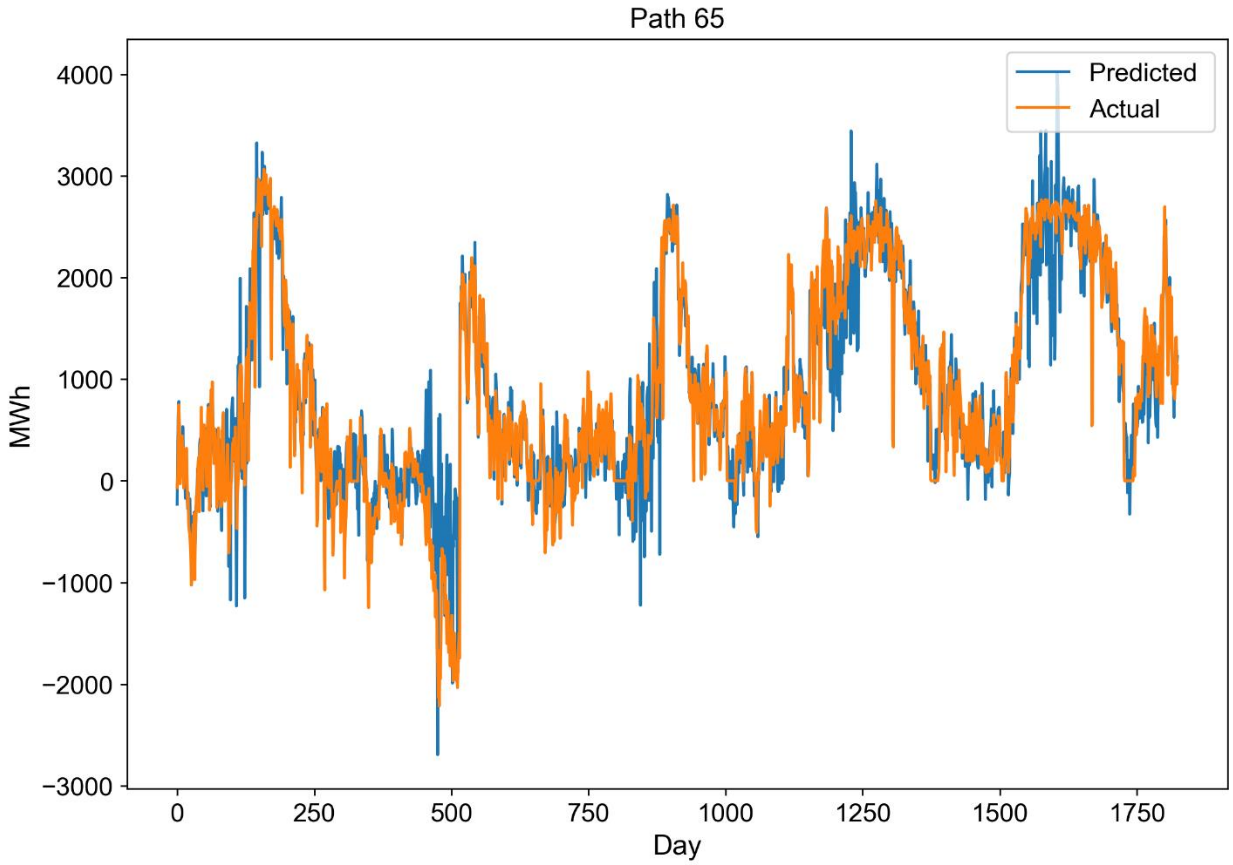

Figure S21. Actual power transfer through Path 65 for the 2010-2012 vs. CAPOW prediction of power flow for the same period (R^2 = 0.85)

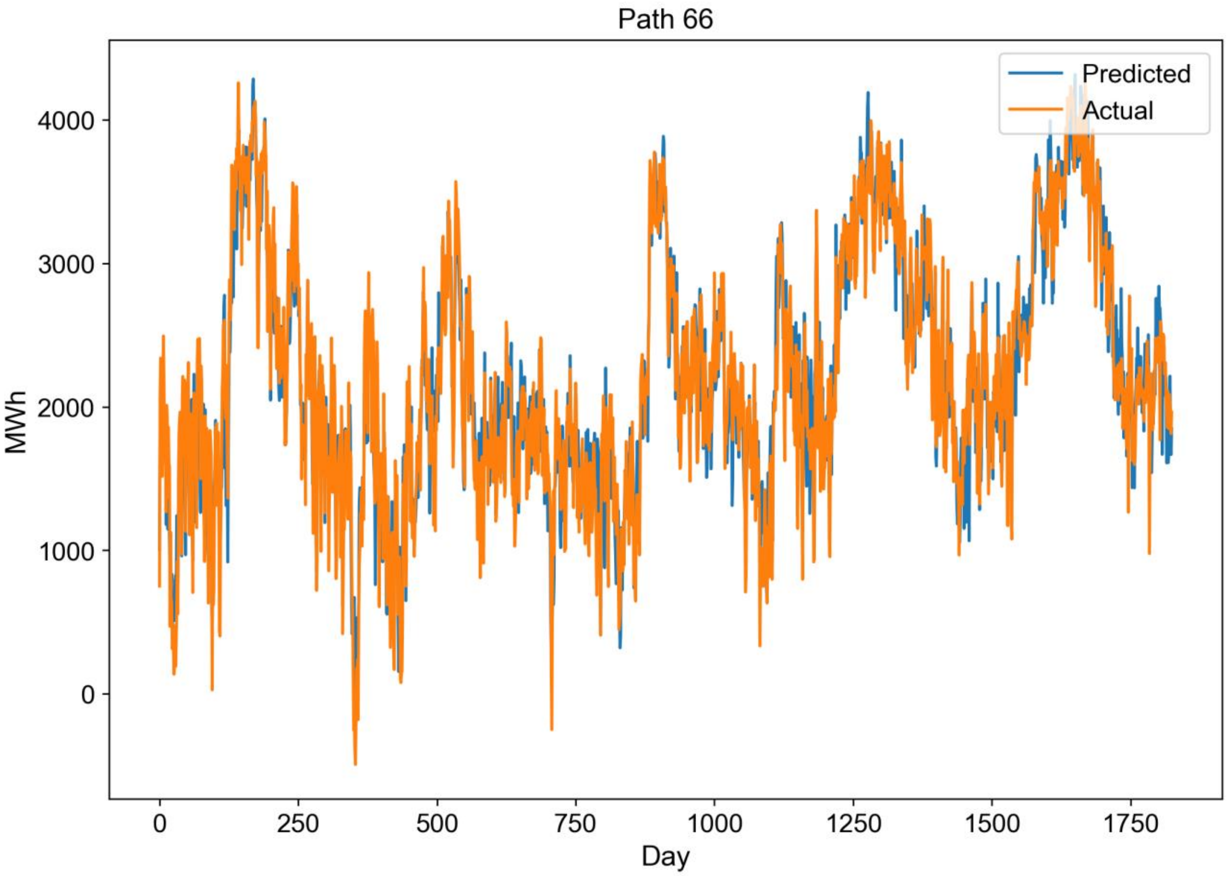

Figure S22. Actual power transfer through Path 66 for the 2010-2012 vs. CAPOW prediction of power flow for the same period (R^2 = 0.89)

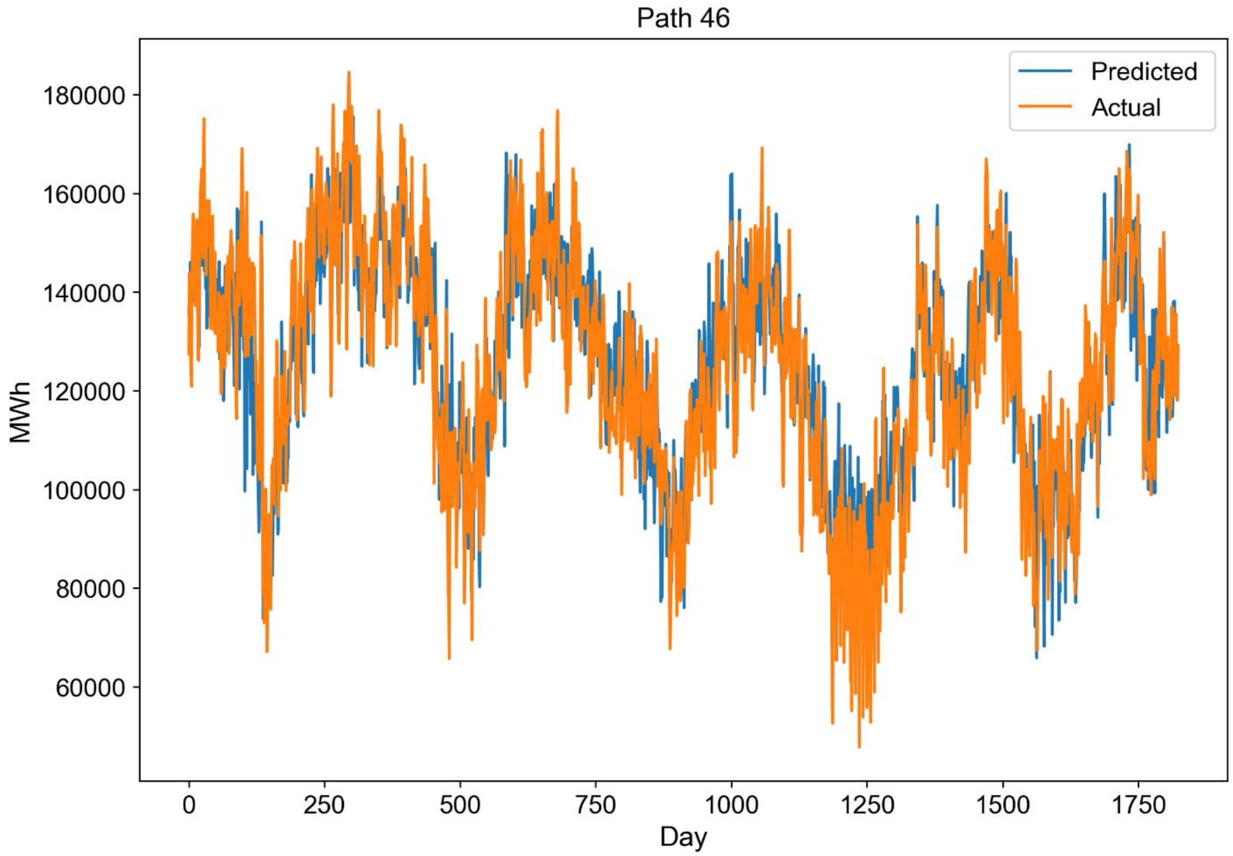

Figure S23. Actual power transfer through Path 46 for the 2010-2012 vs. CAPOW prediction of power flow for the same period (R^2 = 0.76)

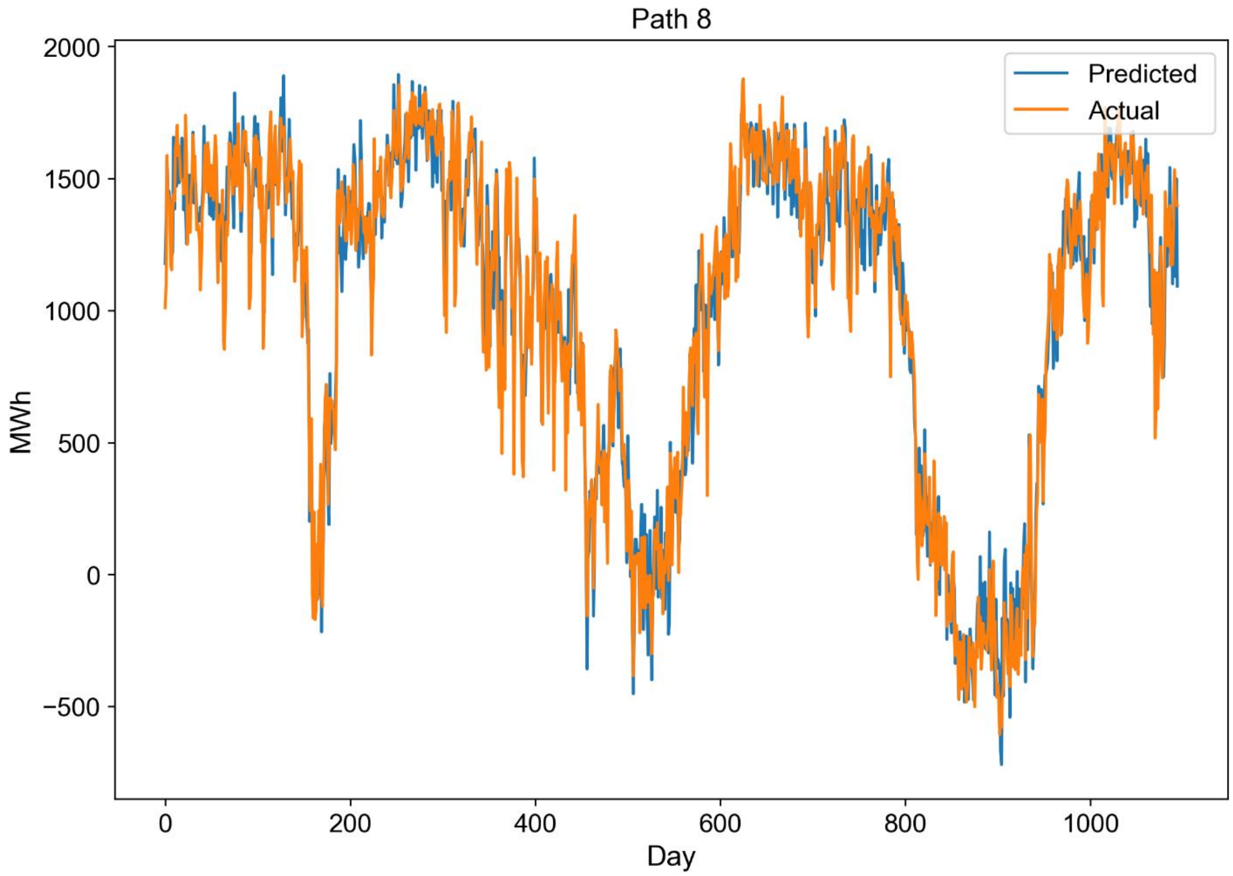

Figure S24. Actual power transfer through Path 8 for the 2010-2012 vs. CAPOW prediction of power flow for the same period (R^2 = 0.83)

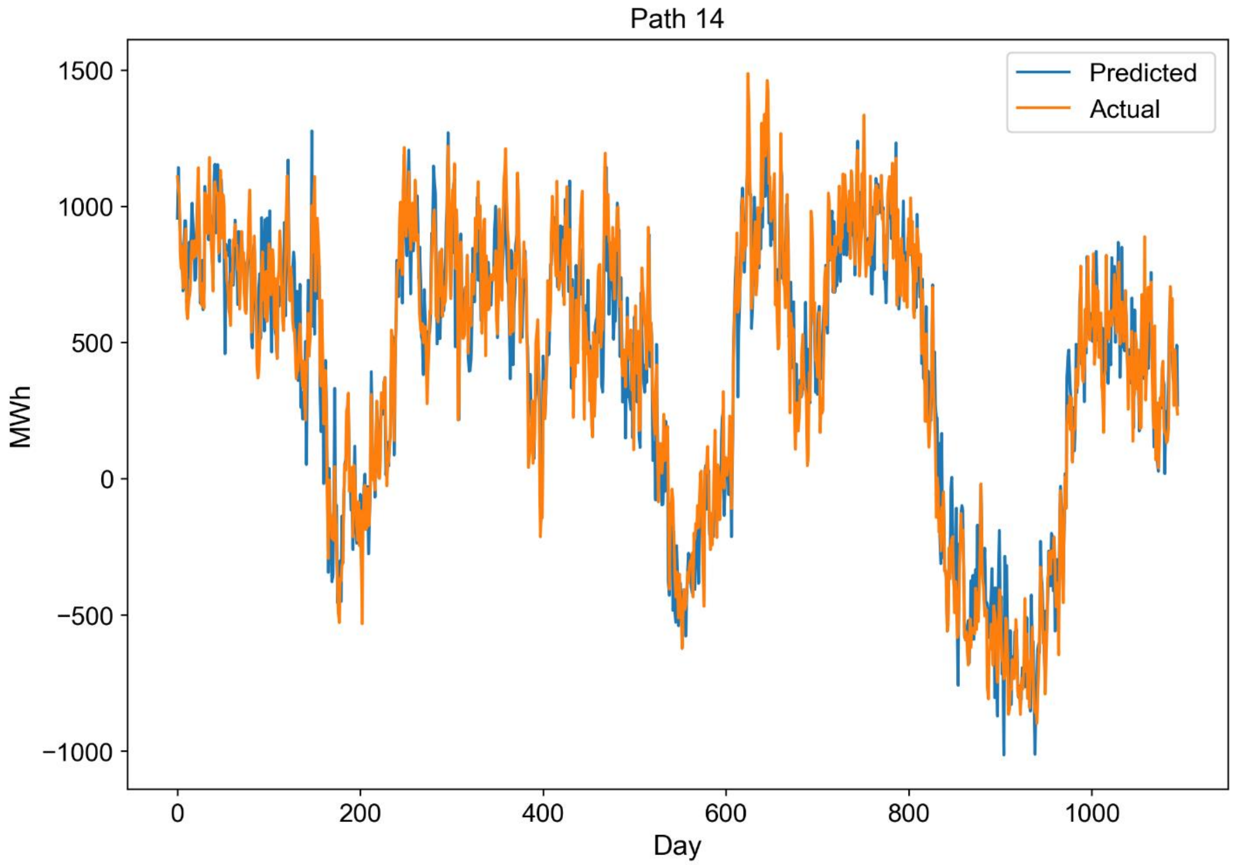

Figure S25. Actual power transfer through Path 14 for the 2010-2012 vs. CAPOW prediction of power flow for the same period (R^2 = 0.79)

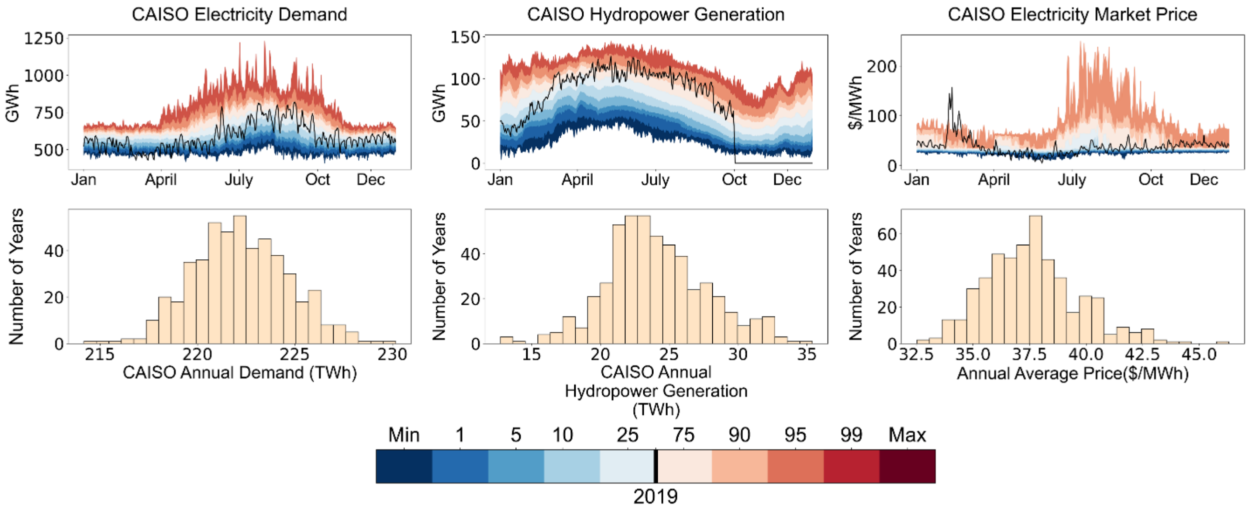

Figure S26. Distribution of CAISO electricity demand, hydropower generation and market price and compare it with the actual data from 2019 from EIA. Historical data shown for electricity demand, market prices and hydropower are shown for 2019. Note that due to missing reported data from EIA from October 2019 to August 2020, the historical hydropower data shown drops to 0 after that point in the calendar year.

1. Su, Y., Kern, J. D., Denaro, S., Hill, J., Reed, P., *et al.* An open source model for quantifying risks in bulk electric power systems from spatially and temporally correlated hydrometeorological processes. *Environmental Modelling and Software* 126, 104667 (2020).
